# DNA Quantity and Quality Comparisons between Cryopreserved and FFPE Tumors from Matched Pan-Cancer Samples

**DOI:** 10.3390/curroncol31050183

**Published:** 2024-04-28

**Authors:** Jeffrey Okojie, Nikole O’Neal, Mackenzie Burr, Peyton Worley, Isaac Packer, DeLaney Anderson, Jack Davis, Bridger Kearns, Kaniz Fatema, Ken Dixon, Jared J. Barrott

**Affiliations:** 1 Department of Cell Biology & Physiology, Brigham Young University, Provo, UT 84602, USA; jokojie@student.byu.edu (J.O.); burrm1@student.byu.edu (M.B.); worleyp@student.byu.edu (P.W.); packatk@student.byu.edu (I.P.); dgrace18@student.byu.edu (D.A.); jackwd@student.byu.edu (J.D.); bkearns9@student.byu.edu (B.K.); 2 Department of Biomedical and Pharmaceutical Sciences, Idaho State University, Pocatello, ID 83209, USA; nikioneal@isu.edu (N.O.); kanizfatema@isu.edu (K.F.); 3 Specicare, 690 Medical Park Ln, Gainesville, GA 30501, USA; 4 Simmons Center for Cancer Research, Brigham Young University, Provo, UT 84602, USA

**Keywords:** biospecimen management, DNA quality, cryopreservation, formalin-fixed, cancer

## Abstract

Personalized cancer care requires molecular characterization of neoplasms. While the research community accepts frozen tissues as the gold standard analyte for molecular assays, the source of tissue for testing in clinical cancer care comes almost universally from formalin-fixed, paraffin-embedded tissue (FFPE). As newer technologies emerge for DNA characterization that requires higher molecular weight DNA, it was necessary to compare the quality of DNA in terms of DNA length between FFPE and cryopreserved samples. We hypothesized that cryopreserved samples would yield higher quantity and superior quality DNA compared to FFPE samples. We analyzed DNA metrics by performing a head-to-head comparison between FFPE and cryopreserved samples from 38 human tumors representing various cancer types. DNA quantity and purity were measured by UV spectrophotometry, and DNA from cryopreserved tissue demonstrated a 4.2-fold increase in DNA yield per mg of tissue (*p*-value < 0.001). DNA quality was measured on a fragment microelectrophoresis analyzer, and again, DNA from cryopreserved tissue demonstrated a 223% increase in the DNA quality number and a 9-fold increase in DNA fragments > 40,000 bp (*p*-value < 0.0001). DNA from the cryopreserved tissues was superior to the DNA from FFPE samples in terms of DNA yield and quality.

## 1. Introduction

With increasing efforts to embrace personalized cancer care, DNA sequencing of tumors and normal tissue is becoming more commonplace. Initiatives, such as the NCI-MATCH and NCI-ComboMATCH trials, which are being spearheaded by the National Cancer Institute, are based on the hypothesis that genomically guided decisions will result in better patient outcomes [1,2]. While there is great promise in molecular characterization of patients’ tumors to guide clinical treatment decisions, the input of the quality of the biospecimen is known to impact the quality of the data output. Formalin fixation was introduced as a clinical preservative in the 1890s and has supported the needs of anatomical pathologists in providing a durable and easily stored biospecimen that can be sectioned, stained, and imaged under a microscope [3]. With the advent of molecular tools for characterizing tumor tissue more thoroughly, molecular pathologists are dependent on a higher quality biospecimen that yields more molecular analytes per milligram of tissue to facilitate multiple molecular assays [4]. Despite evidence that formalin fixation and paraffin embedding (FFPE) damage DNA by fragmenting nucleic acid strands and even introducing mutational artifacts, FFPE tissue is the most prevalent and common practice of processing clinical biospecimens [5,6]. The increased DNA damage in FFPE tissues leads to higher artifacts in sequencing, necessitating a higher tolerance for differentiating between true mutational signals and noise [7]. DNA repair kits and post-sequencing bioinformatics are employed to mitigate these problems. However, they also have their limitations and potential of introducing or overcorrecting identified mutations. Furthermore, next-generation sequencing has a myopic view of the DNA by sequencing < 300 base pair fragments of DNA, thus decreasing the accuracy of identifying structural variants throughout the genome. Long-read sequencing combined with next-generation short-read sequencing can comprehensively identify the entirety of the tumor mutational landscape [8,9,10]. To accomplish this dual sequencing approach, high molecular weight DNA needs to be extracted from the tumor tissue.

This study is a head-to-head comparison between FFPE and cryopreserved-matched tumor samples to determine the impact of formalin fixation and paraffin embedding on key metrics of DNA quantity and quality. The study specifically focuses on the ability to obtain high molecular weight DNA from tumor tissues that can be used by DNA characterization technologies that can accurately identify the structural variants of DNA. The three metrics used to compare DNA between FFPE tissues and cryopreserved tissues are as follows: (1) DNA purity, as determined by the 260/280 ratio, (2) DNA yield, as determined by a UV spectrophotometer, and (3) DNA quality, as determined by DNA fragment microelectrophoresis to assess quantities of DNA at various base pair lengths. We hypothesized that DNA extracted from cryopreserved tissue would be statistically higher in quantity and better in quality when compared to matched samples processed for FFPE.

## 2. Materials and Methods

### 2.1. Sample Procurement

Samples were purchased from Specicare, a commercial biobanking company. Specicare collected tumor tissue from consented patients presenting for surgical removal of tumor tissue during standard of care for malignancy. The samples were split into either formalin-fixed tumor tissue or fresh tumor tissue stored in Hypothermosol solution and transported at 4 °C. Samples were evaluated by a certified board pathologist, and the samples with severe necrosis were considered unacceptable for use in the study. One sample fell into this category and was not included in the dataset. All patient samples were deidentified with unique identification numbers provided by Specicare. Thirty-eight deidentified human patient samples were processed in parallel to be formalin-fixed, embedded in wax or cryopreserved, and stored in liquid nitrogen. Samples were collected between 2018 and 2021. Cryopreserved tissues were stored in LN_2_, and FFPE blocks were stored at room temperature until shipment to Brigham Young University for further analysis.

### 2.2. DNA Extraction

FFPE samples were prepared by cutting multiple 20 μm scrolls of the tissue and dewaxing the tissue in a xylene substitute (VWR, Radnor, PA, USA, 89370-090) followed by ethanol rehydration steps going from 100% ethanol to 100% DI water. The DNA extractions were performed with various starting amounts of tumor tissue ranging from 5 mg to 50 mg. Tissues were weighed after deparaffinization of FFPE scrolls or thawing cryopreserved tissue using the Denver Instrument TP-114. DNA was extracted using an Extracta DNA buffer (Quantabio, Beverly, MA, USA, 95091) combined with proteinase K (Zymo, Dustin, CA, USA, D3001-2) at a final concentration of 1 mg/mL. Tissues were incubated in a cell lysis solution at 55 °C for 3 h in an Incu-shaker Mini (H1001-M). DNA was purified using the Zymo Research DNA Clean and Concentrator-5 Kit (11-302C). The samples were purified to Zymo’s specifications, except 10 μL of DNA Elution Buffer was used in place of the procedural 6 μL. Each centrifugation step was performed using the Eppendorf Centrifuge 5425R set at 21 °C and 14,000 RPM for 60 s.

### 2.3. DNA Concentration and Purity Measurements

Following the extractions, the DNA concentration and purity were measured using a Thermo Scientific Nanodrop One (Waltham, MA, USA, ND-ONEC-W). DNA concentration and purity were calculated using UV absorbance and a ratio between 260 nm and 280 nm.

### 2.4. DNA Fragment Analysis

The DNA fragment analysis was performed on the extracted DNA using the Agilent 5300 Fragment Analyzer System (M5311AA) when analyzing samples of 6000 base pairs and below. For samples with 6000 base pairs or more, the Agilent Femto Pulse System (M5330AA) was used. Samples at 5 ng/μL in 2 μL were prepared using the 55 kb BAC Analysis Kit (Agilent, Santa Clara, CA, USA, FP-1003-0275), and we followed the manufacturer’s recommendations. The gels were fully conditioned, and a prerun of −8.0 kV for 300 s was performed. Samples were injected using −3.0 kV for 15 s and then separated for 90 min. The effect length was 22 cm, and the analyses were performed using the NGS mode on the FEMTO Pulse software version 2.0.0.3. Gels were analyzed for fluorescent intensity and matched to a DNA standard to compare size. Relative fluorescent unit (RFU) is the output to measure the intensity of the band patterns compared to the standard control. The area under the curve was calculated by measuring the RFUs above the baseline after the 62-min mark, which equates to DNA fragment > 40,000 bp.

### 2.5. Statistics

The data were analyzed using two main statistical tests. For the matched-pair tissue samples, a two-tailed student’s *t*-test with equal variance was performed. For the comparison of three or more groups, an Analysis of Variance (ANOVA) test was performed. The data were generated using Microsoft Excel (version 16.84). Statistical significance was deemed as a *p*-value of <0.05.

## 3. Results

### 3.1. Matched Tumor Sample Deidentified Patient Demographics

**Figure 1:**
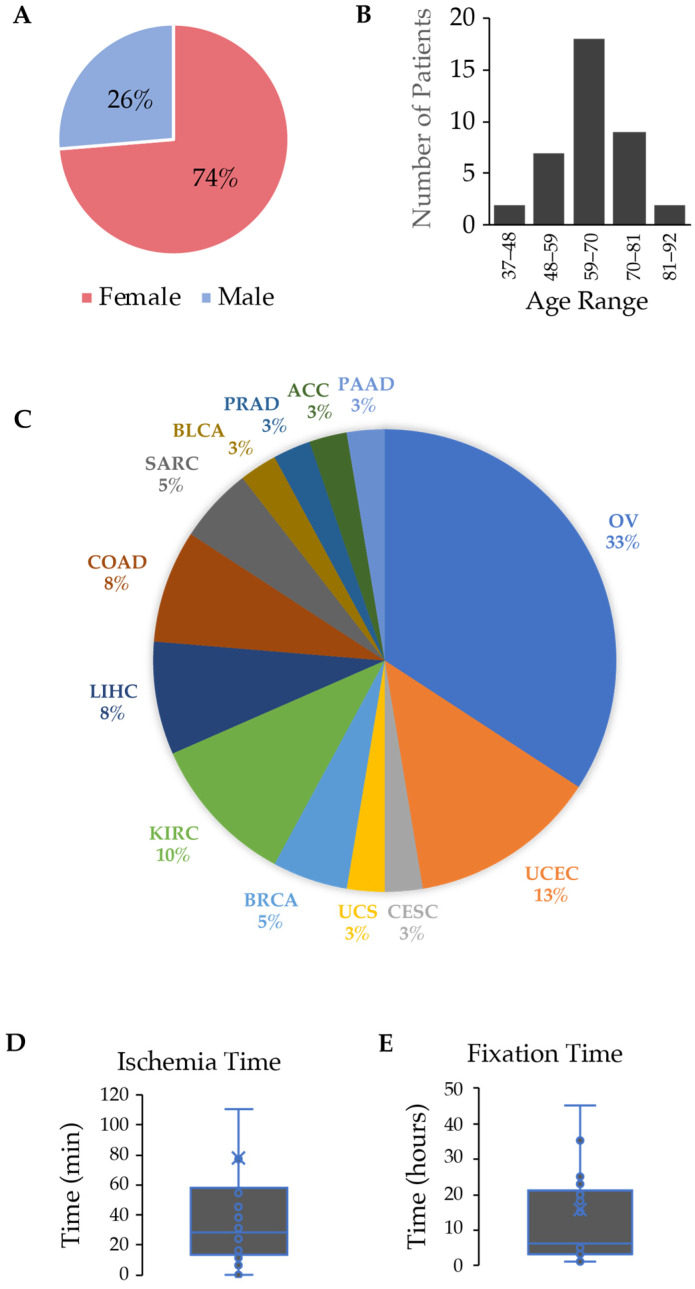
Demographic information about patients (*n* = 38) and the types of tumors included in the study. (**A**) Proportion of female and male patients; (**B**) age range of patients; (**C**) tumor types as indicated by TCGA study abbreviations. ACC, adrenocortical carcinoma; BLCA, bladder urothelial carcinoma; BRCA, breast invasive carcinoma; CESC, cervical squamous cell carcinoma and endocervical adenocarcinoma; COAD, colon adenocarcinoma; KIRC, kidney renal cell carcinoma; LIHC, liver hepatocarcinoma; OV, ovarian serous cystadenocarcinoma; PAAD; pancreatic adenocarcinoma; PRAD, prostate adenocarcinoma; SARC, sarcoma; UCS, uterine carcinosarcoma; UCEC, uterine corpus endometrial carcinoma. (**D**,**E**) Box and whisker plots of ischemia time (minutes) and formalin fixation time (hours), respectively. Outliers were removed, and individual data points (circles) and mean (x) are provided.

Thirty-eight matched tumor samples were obtained from Specicare over a three-year period. Samples were selected randomly to represent a pan-cancer panel of biospecimens to support our approach to analyzing DNA quantity and quality. Samples were deidentified, but demographic information was provided to the researchers along with ischemia times and fixation times in 10% formalin. The sample cohort overrepresented tumors taken from female patients at 74% (Figure 1A); however, the age range (37–86 years of age), median age (65), and distribution more accurately represented pan-cancer demographics (Figure 1B). The cancer types represented by the 38 matched samples are provided in Figure 1C with about half of the cancers falling under the category of gynecological cancers. This explains the overrepresentation of female tumor samples. Ischemia times were controlled across most samples with 30/38 (79%) being less than 60 min before the sample was placed in 10% formalin or a vial of Hypothermosol. The median time was 28.0 min; however, because of some significant outliers (not included in the graph), the mean time was 77.7 min, with a range of 0–828 min (Figure 1D). For fixation times, there were fewer outliers as the protocol was kept more consistent and within the biospecimen management guidelines published by the College of American Pathologists that recommends a minimum of 6 h and a maximum of 72 h fixation time in 10% formalin [11]. The mean fixation time was 15.7 h with one outlier (142 h) not included in the graph (Figure 1E).

### 3.2. Sample Preparation and Purity

Matched samples were prepared for DNA extraction using parallel protocols. FFPE samples were sectioned, dewaxed, and rehydrated in preparation to lyse the cells and extract the DNA. Cryopreserved samples were processed with a more straightforward protocol that involved cell lysis and DNA extraction. Once the samples were lysed and DNA was released from the nuclei, the DNA purification process was identical and employed a DNA binding column to capture DNA and purify DNA through centrifugal wash steps. The fact that the same purification system was used for both tissue types resulted in similar measurements of purity as determined by UV spectrophotometry and calculating the 260 nm to 280 nm ratio for spectral absorbance. A 260/280 absorbance ratio of around 1.8 indicates pure DNA. A ratio outside the range of 1.7–2.0 indicates contaminants [12].

**Figure 2:**
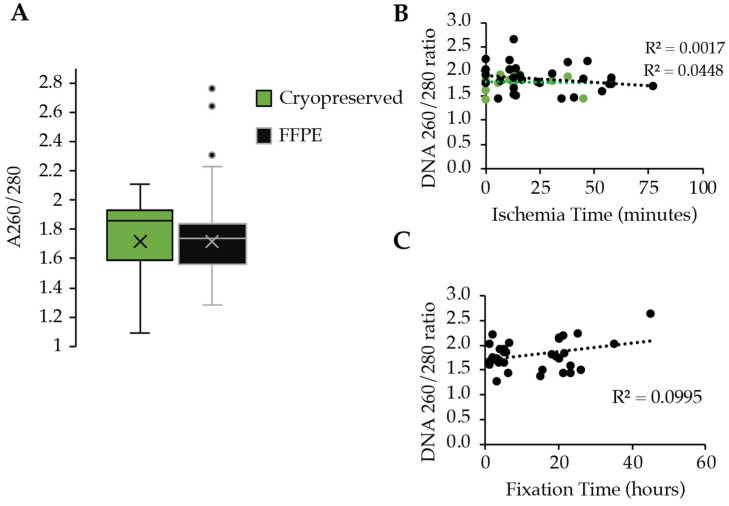
DNA purity comparison between cryopreserved (green) and FFPE (black) tissues. (**A**) Box and whisker plots of the 260/280 ratios of UV absorbance with cryopreserved represented by the green plot and FFPE represented by the black plot; × represents the mean. (**B**) Regression analysis between DNA purity and ischemia time in minutes. (**C**) Regression analysis between DNA purity and fixation time in hours.

In the analyzed DNA samples, the 260/280 ratios of the cryopreserved and the FFPE DNA samples were similar. The mean of the cryopreserved samples (*n* = 74), which included replicates of tumor tissue at various starting masses, was 1.76 ± 0.26 SDM, and the mean of the FFPE samples (*n* = 43) was 1.78 ± 0.32 SDM (Figure 2). Both sample types exhibited acceptable 260/280 absorbance ratios, indicating that pure DNA was being evaluated for further downstream analyses. While the means were almost 1.8 there were samples that fell outside the range of what is acceptable. The higher values in the FFPE samples could indicate the presence of protein or RNA that were still crosslinked to the DNA. There were 7 samples that ranged from 2.15 to 2.76 ratios, while the cryopreserved samples exhibited no samples that were >2.1.

A correlation between DNA purity and ischemia times and fixation times was performed by linear regression analysis. Both cryopreserved and FFPE tissue were analyzed for purity and correlated to the corresponding ischemia time, whereas only the FFPE samples could be correlated to the fixation time and its impact on DNA purity. The low R^2^ values and horizontal slope in the regression analysis indicate that the ischemia and fixation times of the tissues had no significant impact on the purity of the DNA (Figure 2B,C).

### 3.3. DNA Extraction and Yield

**Figure 3:**
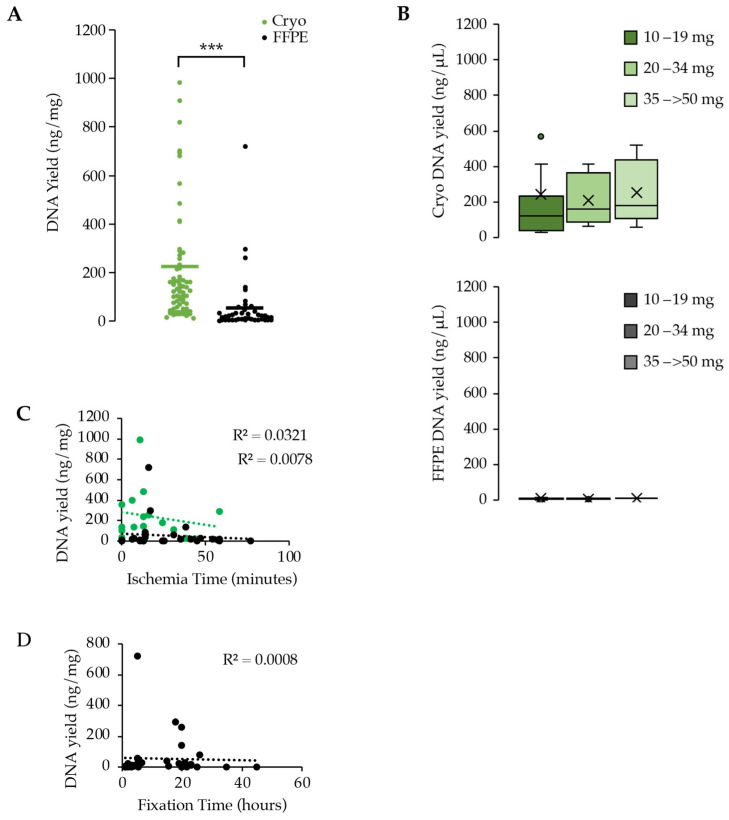
Comparison of DNA yield between cryopreserved samples (green) and FFPE samples (black). (**A**) Combined dot plot demonstrating the yield of DNA (ng) per milligram of tissue; (**B**) box and whisker plots showing the comparison of DNA yield (ng/µL) between different starting amounts of tumor mass and comparing the cryopreserved samples to the FFPE samples; × represents the mean; (**C**) regression analysis between DNA yield and ischemia time in minutes; (**D**) regression analysis between DNA yield and fixation time in hours. *** signifies *p*-value < 0.0001.

After measuring similar values in DNA purity between the cryopreserved tissue and the FFPE tissue, we next evaluated the yield of DNA between the two biospecimen types. Previous studies have addressed the difference between FFPE tissue and flash-frozen (FF) tissue and demonstrated that FF tissue produced 4–10 times the amount of DNA when compared to FFPE [13,14]. The focus of this paper was the comparison between cryopreserved tissue and FFPE, which has less data to support conclusions about a comparison. The DNA from cryopreserved tissue demonstrated an average DNA yield of 222.1 ± 35.2 ng/mg compared to 52.8 ± 18.5 ng/mg displayed in FFPE tissue (*p*-value < 0.001) (Figure 3A). Additionally, the cryopreserved DNA demonstrated an increase in the concentration of DNA that was dependent on the starting amount of tumor tissue compared to FFPE concentrations (Figure 3B). Cryopreserved DNA yielded 238.1 ± 49.5 ng/µL of DNA compared to the significantly lower concentrations of FFPE DNA at 6.2 ± 0.6 ng/µL of tissue (*p*-value < 0.0001).

Using a regression analysis to determine the relationship between DNA yield and ischemia and fixation times, we plotted the values and observed no relationship between the ischemia time or the fixation time and the yield of DNA extracted from the tissue regardless of whether it was cryopreserved tissue or FFPE tissue (Figure 3C,D).

### 3.4. Assessment of DNA Quality between Cryopreserved and FFPE

**Figure 4:**
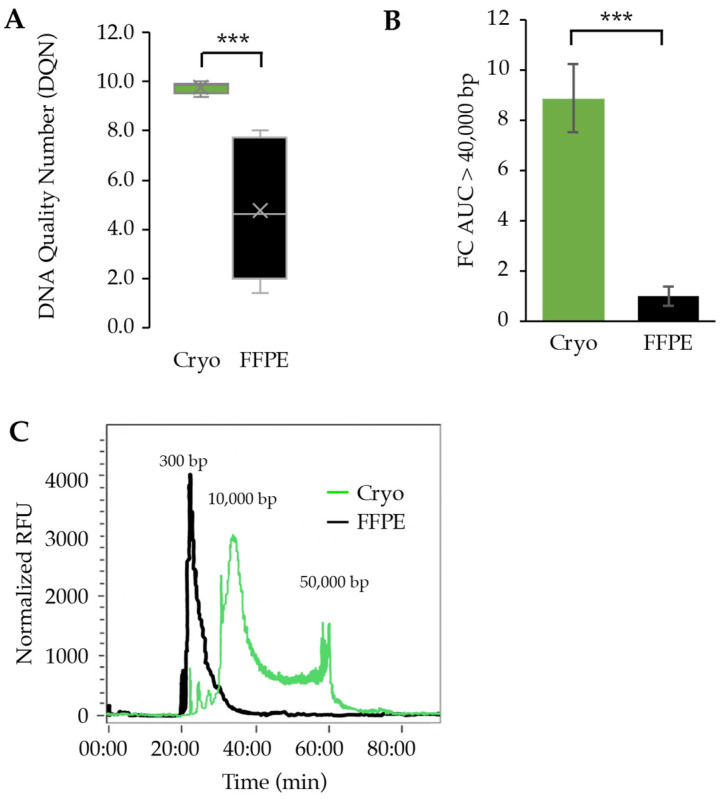
Assessment of DNA quality by fragment analysis comparing cryopreserved samples (green) and FFPE samples (back). (**A**) DNA quality number scores comparisons presented by box and whisker plots; × represents the mean; (**B**) fold change (FC) comparison of the area under the curve (AUC) for fragments > 40,000 base pairs; (**C**) electropherogram of representative samples of DNA from cryopreserved tissue and DNA from FFPE tissue. The relative fluorescent unit (RFU) indicates the quantity of DNA, and the time on the *X*-axis indicates the size of the DNA fragment. ***, *p*-value < 0.0005.

While DNA quantity is an important metric to consider when comparing and choosing between types of biospecimen, ultimately the quality of the DNA input determines the success of downstream molecular characterizations. We determined high-quality DNA by assessing the length of DNA fragments. Differences in the quality of DNA extracted from FFPE and cryopreserved samples began with a comparison of the DNA Quality Number (DQN) for each group. The DQN is a score from 0 to 10 given to a DNA sample that indicates the percentage of DNA fragments that exceed a base pair threshold. A score of 10 corresponds with 100% of fragments meeting or exceeding the threshold, while a score of 0 means that no fragments reached the threshold. The threshold can be arbitrarily chosen, and we set the threshold at 300 base pairs. This is a low threshold for fragment size, but this is the standard size that is needed for next-generation short-read sequencing. The graphs display a stark contrast in DQN scores for the samples that were measured; cryopreserved samples yielded a mean DQN of 9.8 while FFPE had a mean of 4.4 (Figure 4A) with one FFPE sample that returned an “NA” value. The difference in means was statistically significant with a *p*-value < 0.0005. By this measurement, the samples preserved in FFPE provided DNA fragments that were much lower quality than cryopreserved samples.

A secondary perspective of the quality of the DNA extracted came from the analysis of the electropherograms. Electropherograms graphically show the prevalence of DNA fragments of varying lengths after microelectrophoresis separation. The band patterns of DNA are imaged, and the intensity of the bands is used to generate the area under the curve corresponding to the number of fragments with that length present in a sample (Supplemental Figure S1). These graphs were used to compare the number of DNA fragments for each sample that were above 40,000 base pairs, which occurred around the 62:00 time mark. AUC values were normalized based on the concentration (pg/uL) of each sample to ensure equal comparisons. Those values were then normalized again to set the FFPE mean as a base of 1 to compare the cryopreserved values more easily to the FFPE values. After normalization, the cryopreserved tissues yielded a mean AUC above 40,000 bp that was 8.9 times larger than the AUC above 40,000 bp in FFPE tissues (Figure 4B). This means that cryopreserved samples provided almost nine times more fragments above 40,000 bp than FFPE. Further analysis yielded a *p*-value < 0.00005 for a significant difference between the AUC for cryopreserved and FFPE.

The quality difference in DNA fragments extracted from cryopreserved samples versus FFPE samples can be seen in the representative electropherogram in Figure 4C; additional electropherograms with their corresponding DQN can be viewed in the supplemental figures (Supplemental Figure S2). The graph indicates the time at which DNA fragments elute when run through gel electrophoresis, with larger fragments of DNA taking longer to elute than shorter fragments. FFPE (black) has one clear peak towards the beginning of the graph around 22 min, showing a high concentration of small fragments of DNA (approximately 100–400 bp). The distribution of cryopreserved (green) is bimodal, with a large peak around 35 min and another at 60 min (>50,000 bp). Cryopreserved tissues produced higher molecular weight DNA, with many taking up to 60 min to elute, while the majority of fragments from FFPE eluted by the 25th minute (Figure 4C).

## 4. Discussion

In this study, we extracted DNA from FFPE and cryopreserved patient samples and analyzed them using three criteria: purity, yield, and quality. The purpose of this study was to compare DNA from FFPE and cryopreserved tissue samples. FFPE samples are considered the gold standard in the preservation of human tumor tissues, primarily due to their ease of storage and applications in diagnostics for pathologists [15,16]. Recently with the focus on genetic testing, there has been an emphasis on using fresh, frozen, or cryopreserved tissue over FFPE tissue due to the process of formalin fixation and embedding leading to chemical crosslinking, fragmentation, and degradation of DNA molecules. Additionally, the formalin fixation process can result in the deamination of cytosine to form thymine and guanine to form adenine [16,17]. These artifactual changes in DNA base pairs could misguide diagnoses and treatment decisions in the age of precision medicine.

Our study strongly supports the use of cryopreserved tissue to perform molecular characterization on DNA over FFPE tissue. This is poignantly evident in the fragment analysis that demonstrated a stark contrast between DNA extracted from cryopreserved tissue and DNA extracted from matched FFPE tissue. This is becoming increasingly important in newer technologies that depend on high molecular weight DNA for analysis, such as long-read sequencing and optical genome mapping [8,18]. Protocols for these applications can generate even longer DNA fragments ranging from 30 kb to 2000 kb [19,20,21]. High molecular weight DNA allows for more precise detection of large structural variants that are often associated with tumors that are genomically unstable [9,22]. These structural variants, such as chromosomal translocations, can be used for diagnostics and identification of molecular targets for precision therapies but are a missed opportunity because of the reliance of genomic information on FFPE tissue. Additionally, the identification of structural variants as potential neoantigens in tumors remains largely untapped due to the current DNA extraction protocols that result in DNA fragmentation and sequencing that focuses on short DNA fragments (<300 bp). However, sophisticated algorithms can be employed using short-read sequencing data to capture structural variants [23], but this is based on general assumptions seen in a small sample size and a few cancer types. Furthermore, scientists still argue that the overall concordance across whole exomes or genomes is high enough between frozen tissue and FFPE, thus dismissing the need to use frozen tissue over FFPE [24]. While that may be true across thousands of loci in an entire genome, specific oncogenes and tumor suppressor genes have demonstrated discordance rates as high as 27% [25,26,27]. Unfortunately, this study has yet to generate the sequencing needed to show concordance between cryopreserved samples and FFPE samples in support of the downstream sequencing benefits of cryopreserved tissue. Another way to overcome the fragmentation or artefactual mutations in FFPE tissue is to perform sequencing at a depth of >1000 reads per base. This is the method for FDA-approved cancer gene panels when sequencing DNA from FFPE tissue.

In the current study, we used a Femto Pulse to analyze the fragment size of the DNA, which limited our ability to detect even higher molecular weight DNA. For most of the DNA extracted from cryopreserved samples, we observed a bimodal peak distribution around 10,000 bp and 50,000 bp. This can be attributed to the length of the capillary tube, and if it were longer, we posit that the DNA fragment distribution would show an extended tail to the right, indicating those fragments that likely exceed >100 kb. Instead, all the high molecular weight DNA exited at the end of the capillary tube at the same time, which is the explanation for the second peak at the end of the run (Figure 4C).

While we were not surprised to find a difference between the DQN scores between the cryopreserved tissue and the FFPE tissue, it was astonishing to observe how low the DQN scores were for the FFPE tissue. While the cryopreserved tissue demonstrated a 98% success rate of meeting the low threshold of 300 bp, only 44% of the FFPE samples successfully achieved that mark. Over half of the DNA extracted from FFPE tissues is so fragmented that it is not suitable for short-read next-generation sequencing. This is problematic for a tissue type that we demonstrated already yields less DNA per milligram of tissue to begin with. Our results are comparable to others who have measured DNA quality in FFPE tissues. In a large breast cancer study where 1859 FFPE samples were analyzed, DNA Integrity Number (DIN) was found to have a median of 3.8 [28]. In another smaller study comparing FF and FFPE samples, kidney cancer and normal tissue were prepared and DNA was extracted in preparation for bisulfite sequencing; however, the FFPE tissue exhibited a DIN of 1.6 for both cancer and normal kidney tissue, whereas the FF tissue exhibited DIN scores of 9.8 and 9.6, respectively [29]. DIN and DQN are slightly different ways of assessing DNA quality, but both correlate with DNA size and are used for quality control measurements for DNA.

The patients that benefit most from molecular analysis of their tumors are the ones with recurrent or metastatic disease. Oftentimes, patients who present with recurrent or metastatic disease do not undergo resection of the tumor and are relegated to needle biopsies to procure tissue material, which will net 5–20 mg of tissue [30]. As we observed in our study, 5 mg of tissue taken from FFPE on average will yield 250 ng total of DNA, and based on the DQN scores, less than half is of sufficient length to perform next-generation sequencing without amplification. Conversely, 5 mg of tissue taken from cryopreserved tissue will yield, on average, 1250 ng total DNA with DQN scores that suggest that 98% is usable for next-generation sequencing and fragments are large enough for downstream applications to accurately characterize structural variants and genomic instability.

DNA extracted from a core needle biopsy that has been cryopreserved has the potential to generate sufficient quality and quantity of DNA not only for next-generation sequencing but to allow for multiomic analyses on a given tumor. While we have focused exclusively on DNA quality and yield, the process of formalin fixation and paraffin embedding has the same detrimental consequences on other bioanalytes, including RNA, protein, and DNA methylation [27,31,32,33,34]. One variable that was not tightly controlled in our study was the ischemia time and fixation time. There was some variability, as demonstrated in the graph in Figure 1D for the ischemia time, but that would equally impact the FFPE and cryopreserved samples. Regarding the fixation times found in Figure 1E, it could be argued that inconsistent processing could lead to an impact on the DNA integrity. This argument underlines the significance of either establishing fixed guidelines or switching to an alternative sample storage format that has less dependency on processing protocols and fixation times. Despite the variability in the pre-extraction processing, most samples conformed with CAP guidelines established for processing and fixing breast tissue that recommend less than 60 min of ischemia time and 6 to 24 h of fixation time [11]. Further correlation analysis demonstrated that the processing steps that included ischemia time and fixation time for FFPE samples did not impact DNA purity and yield.

While most previous articles have focused on the comparison between fresh frozen (FF) and FFPE tissue [15,17,26,34,35], we have deviated slightly to focus on the comparison between cryopreserved tissue and FFPE tissue. The field largely accepts that the quality of DNA between FF and cryopreserved tissue is the same, and one study has demonstrated the equivalence of DNA between FF and cryopreserved tissue. However, the area where cryopreserved tissue excels is in downstream live tissue testing where viable cells are required [36]. Advances in precision medicine rely on patient-derived tissue to perform ex vivo drug testing, xenograft modeling, and 3D organoid modeling, and the most viable source of tissue is either fresh from the patient or through a slow freezing, cryopreservation method [37], as was used in this study.

Therefore, to maximize the amount of information and the quality of information obtained from tumor biospecimens, it is imperative that methods for biospecimen procurement and storage be improved in clinical practice. Our data support and corroborate the literature in that fresh, cryopreserved, and flash-frozen are superior methods of biospecimen storage when compared to FFPE in the age of molecular diagnostics.

## 5. Conclusions

Cryopreserved cancer tissue provides superior quality assurance measurements of DNA over FFPE. Treatment decisions based on molecular results demand accuracy and validity. The medical community should support efforts to cryopreserve cancer biospecimens in the clinical setting to provide valid molecular testing results. The automatic processing of tumor specimens in formalin is no longer an acceptable default.

## Data Availability

No datasets were generated because of this study. Summaries for all data are provided in the manuscript. Raw data can be obtained by contacting the corresponding author.

## Supplementary Materials

The following supporting information can be downloaded at: https://www.mdpi.com/article/10.3390/curroncol31050183/s1, Figure S1: DNA gel images comparing cryopreserved samples and FFPE samples. Figure S2: Electropherograms of extracted DNA from cryopreserved and FFPE samples.
