# Supplementary material for: DNA Quantity and Quality Comparisons between Cryopreserved and FFPE Tumors from Matched Pan-Cancer Samples
Source: Curr Oncol. 2024 Apr 28;31(5):2441–52. doi: 10.3390/curroncol31050183 (PMC11119490; doi:10.3390/curroncol31050183)
Supplement: Supplementary file 1 [file curroncol-31-00183-s001.zip › curroncol-2945918-supplementary.pdf]

Supplemental Figure S1

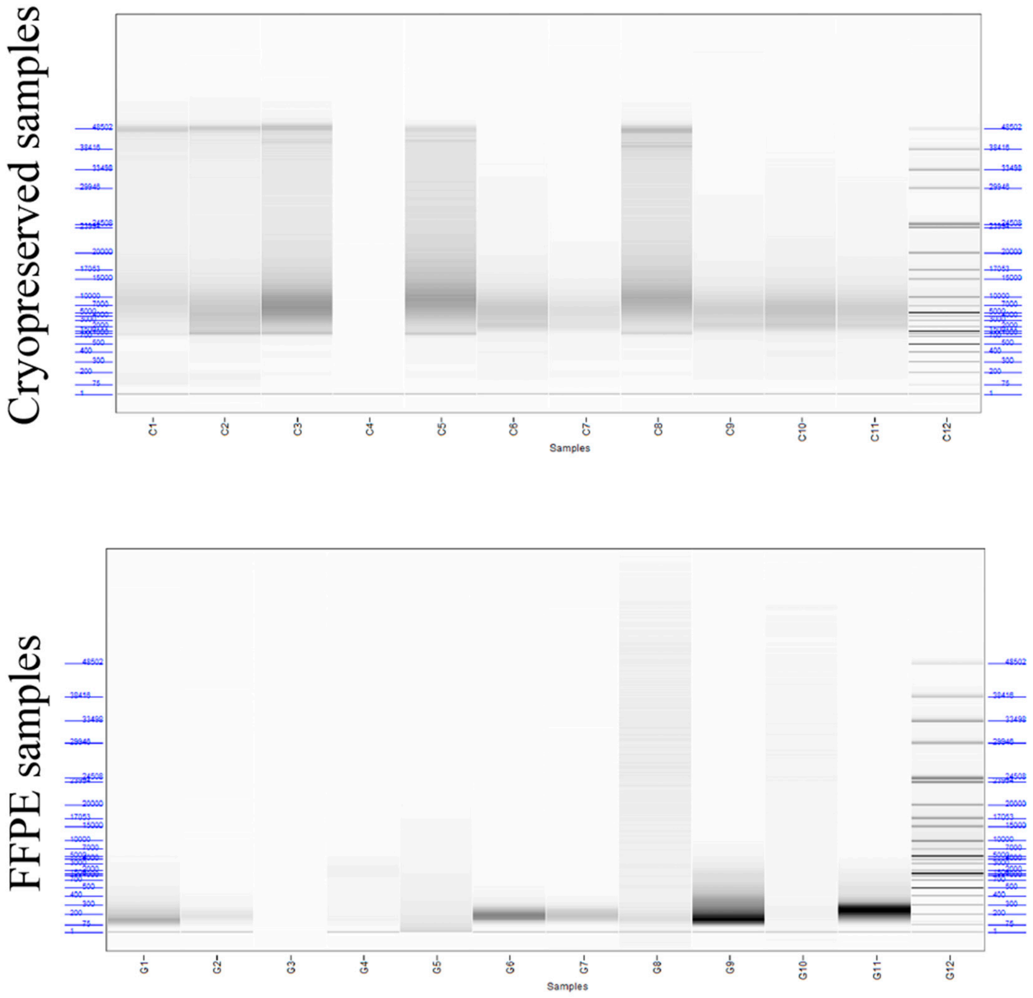

Supplemental Figure S1. DNA gel images comparing cryopreserved samples and FFPE samples. 11 samples shown for each. The far right lane is the standard ladder ranging from 1 bp to 48,502 bp.

Supplemental Figure S2

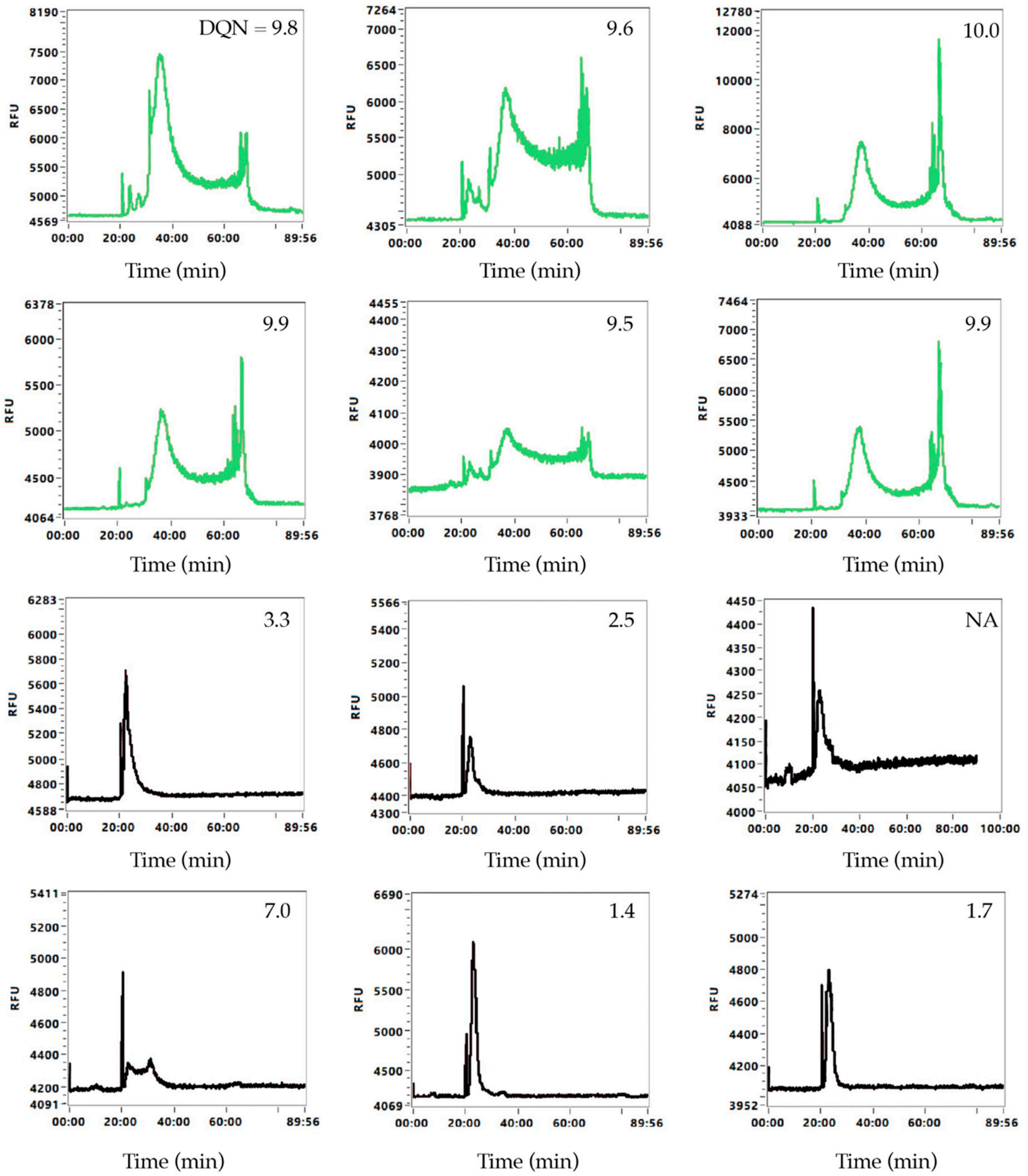

Supplemental Figure S2. Extended examples of electropherograms for cryopreserved samples (green) and FFPE samples (black). These graphs were used to calculate the AUC > 40,000 bp (62:00). The DNA quality number (DQN) is provided in the upper right corner of each graph.
